# Chemistry and Toxicology of Major Bioactive Substances in *Inocybe* Mushrooms

**DOI:** 10.3390/ijms22042218

**Published:** 2021-02-23

**Authors:** Jiri Patocka, Ran Wu, Eugenie Nepovimova, Martin Valis, Wenda Wu, Kamil Kuca

**Affiliations:** 1 Department of Radiology, Toxicology and Civil Protection, Faculty of Health and Social Studies, University of South Bohemia, 37005 Ceske Budejovice, Czech Republic; toxicology@toxicology.cz; 2 Biomedical Research Centre, University Hospital, 50003 Hradec Kralove, Czech Republic; 3 MOE Joint International Research Laboratory of Animal Health and Food Safety, College of Veterinary Medicine, Nanjing Agricultural University, Nanjing 210095, China; wuranvicky@163.com; 4 Department of Chemistry, Faculty of Science, University of Hradec Králové, Rokitanského 62, 500 03 Hradec Kralove, Czech Republic; eugenie.nepovimova@uhk.cz; 5 Department of Neurology of the Medical Faculty of Charles University and University Hospital in Hradec Kralove, Sokolska 581, 50005 Hradec Kralove, Czech Republic; martin.valis@fnhk.cz

**Keywords:** *Inocybe* mushroom, muscarine, psilocybin, psilocin, toxicology

## Abstract

Mushroom poisoning has always been a threat to human health. There are a large number of reports about ingestion of poisonous mushrooms every year around the world. It attracts the attention of researchers, especially in the aspects of toxin composition, toxic mechanism and toxin application in poisonous mushroom. *Inocybe* is a large genus of mushrooms and contains toxic substances including muscarine, psilocybin, psilocin, aeruginascin, lectins and baeocystin. In order to prevent and remedy mushroom poisoning, it is significant to clarify the toxic effects and mechanisms of these bioactive substances. In this review article, we summarize the chemistry, most known toxic effects and mechanisms of major toxic substances in *Inocybe* mushrooms, especially muscarine, psilocybin and psilocin. Their available toxicity data (different species, different administration routes) published formerly are also summarized. In addition, the treatment and medical application of these toxic substances in *Inocybe* mushrooms are also discussed. We hope that this review will help understanding of the chemistry and toxicology of *Inocybe* mushrooms as well as the potential clinical application of its bioactive substances to benefit human beings.

## 1. Introduction

Poisoning incidents caused by ingesting poisonous mushrooms occur every year all over the world, and a large number of poisoning incidents have been reported. Accidental poisoning by foraged mushrooms can affect health or even lead to death in some cases. Mushroom poisoning is a worldwide food safety issue and it has been one of the main causes of food poisoning deaths.

In recent years, more than 100 kinds of poisonous mushrooms have been found, of which more than 30 have fatal toxicity. Among the many poisonous mushrooms, the genus *Inocybe* are common poisonous mushrooms. Many species of the genus *Inocybe*, a highly diverse genus of ectomycorrhizal Agaricales, are world-wide mycorrhizal mushrooms. At present, we mainly distinguish edible fungi and toadstools by morphology, but *Inocybe* mushrooms are often confused with edible species because of their similar appearance, which is also often the cause of mushroom poisoning [1]. People and animals may show some toxic symptoms after ingesting these mushrooms. The main toxic substances of these fungi are muscarine, psilocybin and psilocin. They act primarily on the central nervous system and may cause a range of neurological symptoms. Muscarine increases parasympathetic tone by M-type of post-ganglionic parasympathetic receptors located in muscles and glands, and it causes parasympathetic nervous excitement, heart rate slows down and weakens, muscle contractions, increased gland secretion and pupils become constricted. Furthermore, it can act on the central nervous system and cause nerve excitement [2]. Muscarine is fatal to human beings if too much is ingested at once. According to the available data, the human lethal dose of muscarine in the aforementioned mushrooms is about 500 g of raw mushroom. However, 10 to 20 g of raw mushroom is enough to lead to poisoning [3]. Thus, eating a small number of poisonous mushrooms by mistake is very likely to be poisonous.

Psilocybin and psilocin are neurohallucinogenic toxins and they cause people to experience hallucinations, uncontrollable laughter, extreme excitement and other strange feelings after ingestion. Therefore, they are also known as “laughing mushrooms” and are banned in many western countries as drugs because of these strange neurological symptoms.

Although these toxins have toxic effects and may threaten human life and health, they have been applied to scientific research and biomedicine with the development and maturity of scientific research. Psilocybin and psilocin, particularly, are widely used in the treatment of neurological diseases, drug addiction and psychotherapy due to their mild toxic effects. Psilocybin and psilocin can change the state of neuroconsciousness and reduce anxiety and depression in psychiatric patients. They are also used as a treatment for Alzheimer’s disease, schizophrenia and other mental and psychiatric disorders. Due to their low toxicity and short duration of action, they are often used as a drug substitute for drug treatment to reduce the pain and dependence of drug addicts.

Mushroom poisoning is closely related to people’s health and life, so it is of great significance to understanding toxic components and toxic types of poisonous mushrooms for prevention and treatment of mushroom poisoning. In this review article, we summarized the chemistry, most known toxic effects and mechanisms of major toxic substances in *Inocybe* mushrooms, especially muscarine, psilocybin and psilocin. We also discuss the treatment and applications of these toxins. It is hoped that this review can help us to better understand the main bioactive substances and the clinical application of *Inocybe*, so as to provide some reference for toxin related researchers.

## 2. Mycology

**Figure 1:**
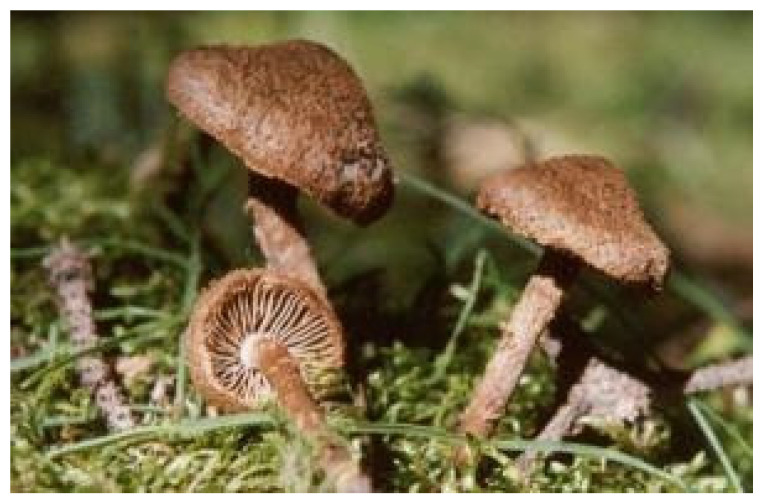
*Inocybe* mushrooms.

*Inocybe* is a large genus of mushrooms in the order Agaricales and family Cortinariaceae (Figure 1). Index Fungorum includes 848 species in *Inocybe* [4]. The genus *Inocybe* (Inocybaceae) has a wide distribution in temperate and tropical regions of the Northern Hemisphere. As for morphological characteristics, the fruiting bodies are small. The cap is deep cinnamon-brown to rust-brown oblate with hairy scales, up to 4 cm in diameter and have margin without cracks. Mushroom meat is white and its fold is straight to nearly extended, light rust color, with white fold margin. The mushroom has a cylindrical almost-white stipe. Its ecological habit is to grow in groups in forests. Ryberg and collaborators considered that large parts of *Inocybe* taxonomy and evolutionary history remain poorly explored [5]. As new species of *Inocybe* mushrooms are discovered, some confusion around this group of mushrooms is quite understandable.

## 3. *Inocybe* Poisoning

*Inocybe* mushrooms contain toxic substances, which can cause poisoning at low dose. Due to the similar appearance to many edible mushrooms, they are often eaten by humans and animals by mistake. Published reports of poisoning with *Inocybe* poisonous fungi are limited to intoxication of dogs and humans.

### 3.1. Animal Poisoning

In general, the number of reported animal poisonings is relatively low. Toxicology testing is only available for a limited number of fungal toxins. Three cases of dog poisoning with *Inocybe* asterospora Quel were recorded in China [6,7], and five cases were recorded in Norway [8]. Common clinical findings in dogs were diarrhea, vomiting, ptyalism and tachycardia. The results of the therapy have shown that the prognosis of *Inocybe* poisoning is very good. All dogs only fully recovered after supportive care.

### 3.2. Human Poisoning

**Figure 2:**
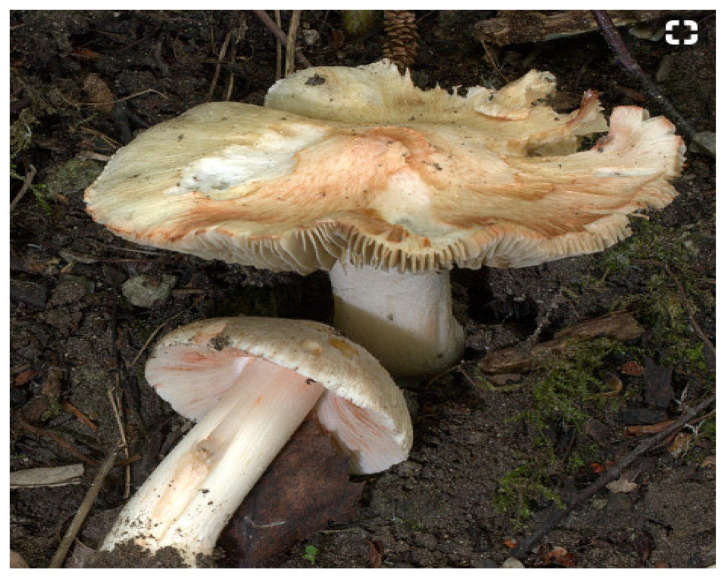
*Inocybe erubescens*, the most common *Inocybe* species associated with toxicity in Europe.

Poisoning by people with *Inocybe* mushrooms is not very common. The diagnosis uses the clinical picture and anamnestic data, mycological and toxicological examination of the residues of mushrooms, their spores and toxins [9]. Several published reports of poisonous *Inocybe* mushrooms were often limited by insufficient identification of the species [1]. *Inocybe erubescens* (syn. *I. patouillardii*) (Figure 2) is the most common type of *Inocybe* associated with toxicity in Europe. Other poisoning of humans was reported in species *Inocybe fastigiata* [10], *Inocybe tristis* [11], *Inocybe asterospora* [7], and *Inocybe aeruginascens* [12].

Although awareness of poisonous mushrooms is increasing, hospitals annually treat many patients diagnosed with poisoning by toxic fungal species. *Inocybe* fungal poisoning has been reported in India in 2015 [13]. A total of 11 people were poisoned, including a 6-month-old child. All those treated were hospitalized. Respiratory distress, vomiting, diarrhea and visual disturbances were observed. It is not known how they were treated, but everyone was released from hospital the next day after intoxication. Tropical *Inocybe carnosibulbosa* CK Pradeep & Matheny, previously unknown in India, have been identified as the cause of poisoning [14]. In 2019 in the province of Ningxia in China, two patients exhibited typical muscarinic symptons after consuming wild mushrooms. The clinical manifestations included chills, sweating, salivation and diarrhea. The incubation period was approximately 2 h. After emergency treatment, they recovered 24 h later. The specimen was identified as *Inocybe serotina* and muscarine was detected [15]. In China, one of the food poisoning accidents in Panyu City, Guangdong province, attracted people’s attention. Ten migrant workers on a construction site ingested mushrooms which grew on cow dung as their dinner. After eating for about 5 min, some people experienced nausea, dizziness, headache and limb weakness. Two hours later, all the workers had the same symptons. The doctor immediately induced emesis, gastrolavage, activated carbon adsorption and other emergency measures; all the workers recovered and were discharged 3 d later. Experts determined that the cause of poisoning was the psilocybin contained in the mushrooms they ate.

The latency to first signs of poisoning varies between 15 min and 2 h after consumption. Clinical manifestations include nausea, vomiting, abdominal pain, diarrhea, hypersalivation, diaphoresis, hypotension, tearing, blurred vision, miosis, tremors, restlessness, flushing and syncope. Additionally, this sickness has a good prognosis after supportive treatment, including intravenous fluids, antiemetics and 1 mg atropine intravenously. Full recovery is usually within 12 h [16].

## 4. *Inocybe* Poisonous Substances

**Figure 3:**
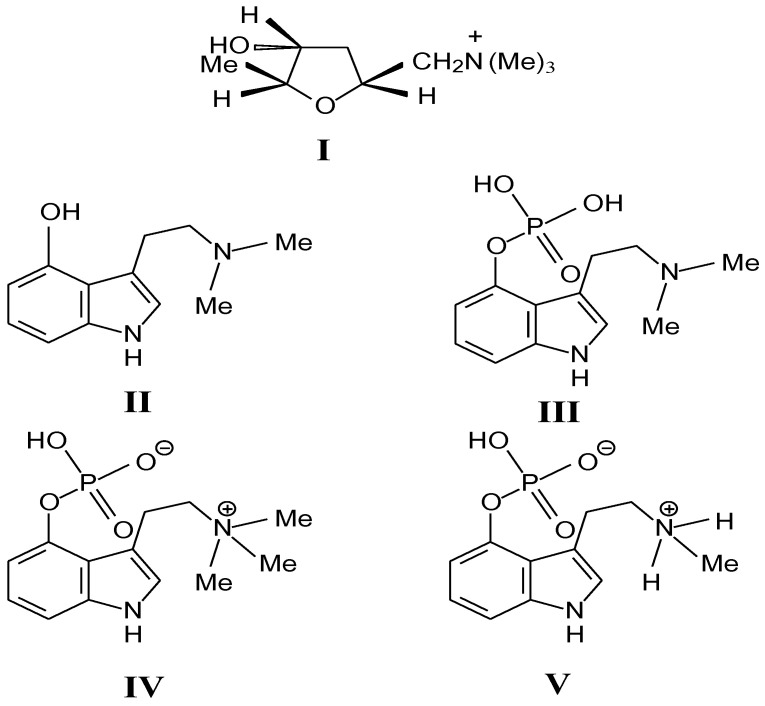
Structures of compounds isolated from the mushrooms of the genus *Inocybe*: I. muscarine; II. psilocin; III. psilocybin; IV. aeruginascin and V. baeocystin.

In order to understand the causes and pathogenesis of human poisoning by ingestion of poisonous mushrooms, many researchers have made great progress in exploring the main toxic substances in mushroom. Based on current knowledge, alkaloids are the main toxic principles of the *Inocybe* mushrooms [17]. The following toxic alkaloids have been found in the *Inocybe* mushrooms: muscarine (I), psilocin (II) and psilocybin (III) and, aeruginascin (IV) and baeocystin (V). The chemical structures of all known *Inocybe*-alkaloids are shown in Figure 3.

### 4.1. Muscarine

Muscarine is named mainly for its isolation from *Amanita muscaria* and is a kind of neurosymptomatic toxin. Muscarine is also found in the genera *Inocybe, Clitocybe, Mycena* and *Omphalotus* [18]. The main poisonous substance of *Inocybe* mushrooms is alkaloid muscarine. The actual muscarine content of *Amanita. muscaria* is only around 0.0003% of the fresh weight [1]. Instead, muscarine concentrations are much higher in *Inocybe* reaching as high as 1.6%. Additionally, the paper chromatography method applied to 34 *Inocybe* species revealed detectable quantities of muscarine ranging from 0.01 to 0.80% in approximately 75% of them [19]. Alkaloid muscarine is a colorless and odorless ammonium quaternary molecule that stimulates the parasympathetic nervous system of animals [20].

The mechanism is similar with acetylcholine, which binds to acetylcholine receptors and generates a characteristic series of symptoms including central nervous system disorder, hallucination-induced inhibition or excitation. Specifically, those include sweating, tearing and bradycardia. These symptoms generally occur rapidly within two hours of eating. Muscarine is particularly widespread in *Inocybe* mushrooms taken from North America and Europe while widespread in east Asia [2,21,22,23]. About their application, some studies have shown that the cholinergic receptor is activated by binding to the muscarine. After activation, it can promote the invasion and metastasis of colon cancer or bile duct cancer cells [24]. That may provide a new therapeutic direction to inhibit tumor growth and metastasis by knocking out the receptor or inhibiting the activation of the receptor, so muscarine has potential clinical value.

#### 4.1.1. Chemistry and Stereochemistry

**Figure 4:**
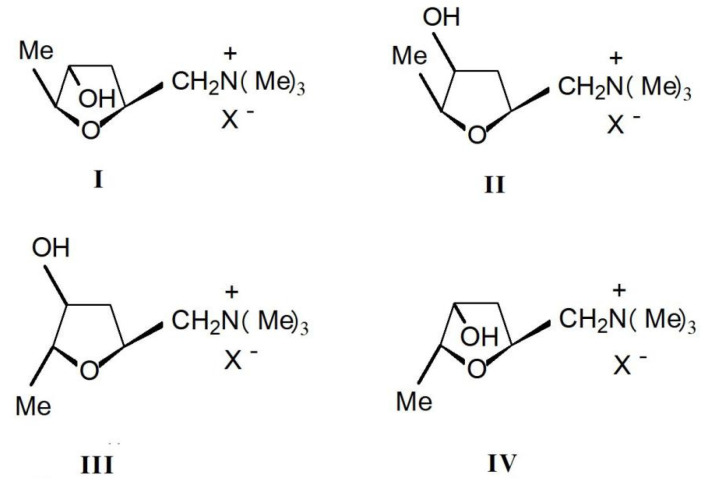
Four stereoisomers of muscarinee: I. muscarine; II.epi-muscarinee; III. allo-muscarinee and IV. epiallo-muscarinee.

Muscarine(L-(+)-muscarinee,(2S,4R,5S)-(4-hydroxy-5-methyl-tetrahydrofuran-2-ylmethyl) trimethylamonium, C_9_H_20_NO_2_, CAS number 300-54-9) is a toxic alkaloid found in fly agaric and other mushrooms of the *Inocybe* species. It is the first parasympathomimetic substance ever studied and causes profound parasympathetic activation that may end in convulsions and death. Muscarinee was firstly isolated and found to exist in four stereoisomers: (+)-muscarinee, (+)-epi-muscarinee, (-)-allo-muscarinee and (+)-epiallo-muscarinee (Figure 4). L-(+)-muscarinee is a natural product found in certain mushrooms, particularly in *Inocybe* and *Clitocybe* species [25]. The distribution and relative concentrations of muscarinee and its stereoisomers in *Inocybe* species were determined by Catalfomo and Eugster. Notable was the finding that *Inocybe cinnamomea*, *Inocybe geophylla*, and *Inocybe lacera* contained concentrations of epi-muscarinee equal to or higher than muscarinee itself [22].

#### 4.1.2. Poisonous Mechanism

The mechanism of muscarinic poisoning is to mimic the action of the neurotransmitter acetylcholine by binding to muscarinic acetylcholine receptors, resulting in continuous impulses of cholinergic nerves [26]. Muscarine acts in the peripheral nervous system, competing with acetylcholine at its receptor binding site. Once bound to the receptor, muscarinic mimics the effects of acetylcholine. Muscarine cannot inactivate acetylcholinesterase, and uncontrolled receptor hyperstimulation occurs. There are five different types of muscarinic receptors: M1–M5, and most tissues express a mixture of subtypes. The M2 and M3 subtypes mediate the muscarinic response of peripheral autonomic tissues. The M1 and M4 subtypes are more abundant in the brain and autonomic ganglia [27]. M1, M3 and M5 interact with Gq protein to stimulate the hydrolysis of phosphoinositide and release intracellular calcium [28]. The M2 and M4 receptors interact with the Gi protein to inhibit adenylate cyclase, resulting in a decrease in the intracellular cyclic adenosine monophosphate concentration. Muscarinic receptors play a leading role in mediating the role of acetylcholine in the brain, and indirectly produce excitatory and inhibitory effects by combining with a series of unique receptor subtypes. Muscarinic receptors are found in both presynapses and postsynapses. Ultimately, their main neuronal effects seem to be mediated by changing the properties of ion channels. Presynaptic muscarinic receptors participate in an important feedback loop that regulates neurotransmitter release [29]. The acetylcholine released from the presynaptic terminal can bind to muscarinic receptors in the same nerve terminal to activate the enzymatic process that regulates subsequent neurotransmitter release.

#### 4.1.3. Clinical Response

Muscarine is a toxic neurotoxic alkaloid that enhances parasympathetic excitability. Muscarinee can cause parasympathetic nervous system disorder around the body and inhibit the role of the acetylcholine in the choline receptor [30]. The symptoms of intoxication with *Inocybe* mushrooms rich in muscarinee are very typical. First, symptoms start early after eating (15–120 min) with headache, nausea and vomiting. Second, salivation and lacrimation follows, combined with miosis, reduced vision and disturbed accommodation. Other signs of poisoning are gastric colic, diarrhea, bronchoconstriction and severe dyspnea. Bradycardia combined with rapid hypotension and vasodilation leads to circulatory shock. Death occurs after approximately 8 h. Usually, muscarine rarely causes death and most of the poisoned people survive without treatment.

#### 4.1.4. Experimental Toxicity

Muscarine is a toxic alkaloid. Published acute and subchronic toxicity parameters of muscarinee are summarized in Table 1.

#### 4.1.5. Human Toxicity

Patients generally recover after muscarineism, but ingestion of large quantities of mushrooms containing the toxin can also cause death. The lethal dose of muscarinee in humans was estimated to be between 180 and 300 mg. Therefore, ingestion of a single mushroom containing 0.33% muscarinee on a dry weight basis can be lethal [38].

#### 4.1.6. Treatment

Effective therapy consists of rapid administration of atropine [39]. Serious poisoning is rare because of the low gastrointestinal bioavailability of muscarinee from the mushrooms and the low concentration of muscarinee. Treatment includes induction of emesis and administration of activated charcoal and rehydration to restore fluid balance and electrolytes is a key component of treatment. If clinical signs of life are present, atropine should be given. After giving a test dose of 0.04 mg/kg (1/4 i.v. and 3/4 i.m. or s.c.) to determine its efficacy, atropine can be given repeatedly until symptoms are abolished or until cessation of salivation [23].

### 4.2. Psilocybin and Psilocin

#### 4.2.1. Chemical Structure

Psilocybin and psilocin are hallucinogenic substances isolated from the fruiting bodies of *Psilocybe mexicana* cultivated by Swiss chemist Hofmann in 1957, and it has been proven that psilocybin and psilocin are kinds of mushroom toxins that can cause neurohallucinogenic effects [40]. These two kinds of toxins are mainly distributed in the genera *Inocybe*, *Agrocybe*, *Conocybe*, *Copelandia*, *Galerina*, *Gymnopilus* and *Hypholoma* [41]. Psilocybin is also known as psilocybine. Its scientific name is 4-phospho-N, N-dimethylamine, which is a small molecule indole alkaloid with hallucinogenic activity. Psilocybin is primarily a pro-drug that is rapidly converted in the body through dephosphorylation by alkaline phosphatase to active metabolite psilocin [42]. Because Psilocybin is unstable in the blood, it is easy to dephosphorylate and exists in the form of psilocybin dephosphorization.

#### 4.2.2. Physico-Chemical Characteristics

Pure psilocybin and psilocin are colorless crystals and sensitive to temperature. When stored for a few months at room temperature, both will be completely deactivated. Whereas, freeze-dried mushrooms will remain active after being stored for more than two years at −5 °C [43]. Cytochrome oxidase oxidizes the dephosphorization of psilocybin to produce the blue product, and hallucinogenic mushrooms that contain the toxin often turn blue after being picked [44]. So, we can identify the hallucinogenic mushroom by this way.

#### 4.2.3. Poisonous Mechanism

The toxicological mechanism of psilocybin is not fully understood. The psychedelic effect of psilocybin is related to the activity of 5-hydroxytryptamine (5-HT), dopamine, epinephrine and other bioamines [45]. In animal pharmacological research data, it was found that dopamine and 5-HT inhibited each other. The imbalance of 5-HT and dopamine in the thalamic striatal cortical margin was a key factor causing neurological symptoms [46]. Electron-ray tomography proved that psilocybin can unbalance 5-HT and dopamine [47]. It has also been suggested that psilocybin poisoning is caused by activation of the 5-HT_2A_ receptor and has nothing to do with dopamine stimulation. Overactivation of the 5-HT_2A_ receptor causes neurological symptoms and inhibition of this receptor leads to a calming effect [48]. The strange hallucinations caused by psilocybin are mainly due to the activation of serotonergic (5-HT_2A_) receptors in the body. Psilocybin induces neural excitatory effects by increasing the receptors of 5-HT_2A_ in the postsynaptic membrane [49].

Psilocin is a mixed serotonergic receptor agonist [50]. It has a high affinity for the 5-HT_2A_ receptor in the brain, where it mimics the effect of serotonin [51]. Psilocin binds less strongly with other serotonergic receptors 5-HT_1A_, 5-HT_2C_ and 5-HT_1D_ [52]. Published acute toxic parameters of psilocin are summarized in Table 2.

#### 4.2.4. Clinical Response

Psychiatric symptoms are associated with dose, environment, previous hallucinogenic experiences, emotions and personality [55]. Psilocybin binds and activates serotonergic (5-HT_2A_) receptors, leading to the physiological and neurotoxic effects. Psilocybin can only cause physiological symptoms at doses of 8–10 mg (0.1–0.2 mg/kg, oral), including mydriasis, light elevated blood pressure, increased heart rate and paresthesia [56,57]. Hallucination occurs when the dose is more than 15 mg [58].

The symptoms of psilocybin poisoning can be divided into three stages. The first stage starts from 20 to 30 min after oral administration and lasts for 10 to 15 min, with implant neurological disorder accompanied by sensory and perceptual interference. The second stage is the peak stage of neurological symptoms, during which the feeling is relatively strong, including personality solution, derealization, spatiotemporal change and weightlessness. Sensory changes mainly include visual disturbances, such as bright and warm colors, especially red and green. Physical sensations including brain vertigo and mental depression are accompanied by anxiety and restlessness. In the final stage, the mental symptoms gradually disappear, while the vegetative nerve interference continues for a period of time. This period can be accompanied by lethargy, extreme exhaustion and mental decadence. In severe cases, delusional syndrome occurs [59].

#### 4.2.5. Treatment

Psilocybin mainly acts on the autonomic nerves, causing nerve excitation, hallucination, tachycardia, dilated pupils and dysuria, but is generally not life-threatening [60]. In general, taking poisonous mushrooms can cause central nervous poisoning (within 20 min) [61]. If poisonous mushrooms are provided in the form of liquid (such as soup), poisoning phenomenon will occur earlier (about 5–10 min) and lasting 2–4 h [62]. Usually, the peak period of poisoning lasts only a little more than an hour [63]. People who have eaten psilocybin mushrooms generally recover completely.

Treatment usually includes induction of emesis and gastrolavage. Vomiting inducing can be used when people ingest psilocybin containing mushrooms within one hour with syrupus ipecacuanhae. However, gastric lavage is generally not used because the stirring produces an unpleasant sensation and whole mushrooms may clog the stomach tube [59]. As for the patients who are in extreme anxiety or have some seriously aggressive behavior, benzodiazepines or chlorpromazine may be taken. When using these anticholinergic agents, respiratory failure should be observed and intubation or pulmonary ventilation devices should be used if necessary [64]. In addition, for most patients, the effective way for them is to rest and recover in a dark and quiet room.

#### 4.2.6. Application

Psilocybin was used in religious practices by native Americans in Central and South America for thousands of years. Psilocybin and psilocin are regulated substances in many countries and are the two major hallucinogenic compounds of “magic mushrooms” [65]. Psilocybin is very popular and often misused.

##### Medical

Although psilocybin has not been widely developed and utilized, a lot of research work has been done on the prevention and treatment of diseases. Psilocybin has been used in various forms for the treatment of mental disorders and addiction since its discovery. Psilocybin is devoted to the scientific community as a research tool for modeling psychosis as well as for possible therapeutic effects [66]. Furthermore, psilocybin can be used in end-of-life care for patients with advanced cancer to ease the pain [67]. Psilocybin is suitable for psychotherapy because it alters consciousness and state of mind and arouses emotions while being low in toxicity. Low-dose psilocybin can be used to treat patients with depression, anxiety and other physical and mental disorders; high dose psilocybin has a definite effect on getting rid of bad habits such as smoking, alcohol and crime [68]. These show the dual value of “psychedelic mushrooms” in both humanities and medicine.

##### Physiological Research

As psilocybin can affect many physiological activities such as hearing, vision, memory and emotion, it is widely used for research basis, formation causes, regulation modes and mutual connections of these physiological activities. It is proven that visual cortex V1 is a structural model composed of interconnected polygonal column units, each of which contains cylinders from many different sources and the lateral connections of these columns should be symmetrical [69]. Carter’s team used psilocybin to explore the relationship between attention and working memory, concluding that attention and working memory are likely to be separate and that there is no obvious link between them [70]. Carter and co-workers conducted binocular vision competition experiment with psilocybin, and the results showed that the tryptamine pathway participated in binocular vision competition [71]. A lot of literatures show that psilocybin and other psychedelics are very helpful for the study of 5-HT receptor subtype [72]. In short, psilocybin is greatly applied to physiological research.

### 4.3. Aeruginascin

Aeruginascin (N,N,N-trimethyl-4-phosphoryloxytryptamine, C_13_H_19_N_2_O_4_P, Mr 298,279 g/mol, CAS Number 114264-95-8) is an indoleamine alkaloid which occurs naturally only within the mushroom *I. aeruginascens* [73]. It is the N-trimethyl analogue of psilocybin [74], and it is closely related to the frog-skin toxin bufotenidine [75].

### 4.4. Baeocystin

Baeocystin, a methyl analogue of psilocybin, is a tryptamine toxin. It was first isolated from the extracts of *Psilocybe baeocystis* [76,77] and *Psilocybe semilanceata* [78,79]. Firstly, it was synthesized by Troxler and his partners in 1959 [80]. Additionally, they are thought to be precursors to psilocybin. We can find that baeocystin is an N-demethylated derivative of psilocybin. The hydrogen on the -OH of one methylpsilocybin is also replaced by phosphoric acid, and the -CH3 is removed from the -N. Additionally, Figure 3 illustrates that the structure of baeocystin (V) exists in its zwitterionic form. The mechanism of baeocystin is similar to psilocybin and psilocin. It can also bind to specific subtypes of the 5-HT receptor to produce hallucinogenic effects [81].

### 4.5. Lectins

Mushrooms are a valuable source of lectins with unique traits and potential for biotechnology and biomedical applications [82]. Mushroom lectins are a significant group of biologically active glycoproteins that have chemopreventive activity against cancer. A new lectin with a molecular weight of 17 kDa and carbohydrate specificity against lactose and galactose has been isolated from *Inocybe umbrinella* dried fruits. This lectin has a unique N-terminal amino acid sequence, DGVLATNAVA, and it is active against hepatocellular carcinoma cell line (HepG2, 3.5 ± 0.2 μmol/L). However, the production of this lectin from fresh mushroom is impractical, because only 15 mg lectin per 100 g fruiting bodies of *I. umbrinella* was isolated [83].

## 5. Conclusions

People have the habit of eating mushrooms because of its delicious taste, rich nutrition and high medicinal value. However, there are so many kinds of mushrooms which look similar. The species diversity of poisonous mushrooms is quite rich. If edible and poisonous mushrooms cannot be identified, it may cause toxic mushroom poisoning and even lead to death. Mushroom poisoning is closely related to people’s health and lives. Mushroom poisoning is still a threat to human life. The incidence of mushroom poisoning is high, the mortality rate is high and the toxins contained in mushrooms are complex and varied. The clinical manifestations of poisoning are varied. Currently, there is no specific antidote for mushroom poisoning, and only medication and blood purification can be given as symptomatic supportive treatment. For instance, toxins found in the genus *Inocybe* show signs of affecting the central nervous system, the parasympathetic system and the gastrointestinal system. The effects of toxins in the above-mentioned mushroom species are generally mild, but the symptoms depend on both the dose of the toxins consumed and the individual sensitivity. In most cases, treatment is symptomatic.

Currently, there is a lack of basic research on mushroom poisoning. Clinical research is mainly focused on the general epidemiology and case reports of poisonous mushrooms, and there is a lack of exploration of toxins and research on the pathogenesis of the toxins. Actually, poisonous mushrooms and their toxins have broad development prospects and they have been used in many aspects such as biological control, anti-cancer medicine and biotechnology. Exploitation and utilization of toxic mushroom resources will benefit people. The genus *Inocybe* is very numerous and new species are still being discovered. So far, toxicological analysis has only been performed on a small number of species, and it can be expected that further unknown biochemicals will be gradually discovered. Therefore, it is assumed that new substances with interesting biological effects will be discovered. With the progress of toxin detection technology, especially the development and application of metabonomic technology, toxins caused by toadstool poisoning will be easier to detect, and more new toxins will be gradually discovered, so as to better study the mechanisms of action of different toxins causing poisoning. At the same time, it is of great help to determine the toxic components, poisoning types and poisoning mechanisms of poisonous mushrooms, and also reduce the harm of poisonous mushrooms to human health.

## Figures and Tables

**Table 1:** Published acute and subacute toxic parameters of muscarinee.

Organism	Test Type	Route	Reported Dose	Literature Source
frog	LDLo	parenteral	0.16 mg/kg	[31]
frog	LDLo	unreported	0.16 mg/kg	[32]
mouse	LD_50_	intraperitoneal	5 mg/kg	[33]
mouse	LD_50_	intravenous	0.23 mg/kg	Toxnet
mouse	LD_50_	subcutaneous	1.1 mg/kg	[34]
mouse	LDLo	oral	750 mg/kg	[35]
rabbit	LDLo	oral	150 mg/kg	Toxnet
rabbit	LDLo	subcutaneous	27 mg/kg	Toxnet
cat	LDLo	intravenous	1.1 mg/kg	[36]
cat	LDLo	oral	7 mg/kg	Toxnet
cat	LDLo	subcutaneous	2 mg/kg	[32]
man	LDLo	unreported	0.735 mg/kg	[37]

LD_50_ = median lethal dose, LDLo = lethal dose low.

**Table 2:** Published acute and subacute toxic parameters of psilocin.

Organism	Test Type	Route	Reported Dose	Literature Source
mouse	LD_50_	intraperitoneal	196 mg/kg	[53]
mouse	LD_50_	intravenous	74 mg/kg	[54]
rat	LD_50_	intravenous	75 mg/kg	Toxnet
rabbit	LD_50_	intravenous	7 mg/kg	[54]

LD_50_ = median lethal dose.

## Data Availability

Not applicable.
